# Scarlatina, Vel Febris Rubra,—or Scarlet Fever

**Published:** 1838-12-05

**Authors:** N. Chapman

**Affiliations:** Professor of the Theory and Practice of Physic, in the University of Pennsylvania


### THE MEDICAL EXAMINER.

DEVOTED TO MEDICINE, SURGERY, AND THE COLLATERAL SCIENCES.

EDITED BY J. B. BIDDLE, M.D., M. CLYMER, M.D., AND W. W. GERHARD, M.D.

No. 25.] PHILADELPHIA, WEDNESDAY, DECEMBER 5, 1838. [Vol. I.

### SCARLATINA, VEL FEBRIS RUBRA,—OR SCARLET FEVER.

*By* N. Chapman, M. D., *Professor of the Theory and Practice of Physic, in the University of Pennsylvania.*

(Concluded from page 381.)

Not much is recorded of the anatomical characters in this disease. It may be collected, however, from the dissections which have been occasionally reported, that the mucous tissue of the primæ viæ, particularly of the stomach and upper intestines, is highly inflamed, and scarcely less frequently are the lungs, with the brain, in their substance and membranes, in the same condition. It ought to be added, that some recent researches have revealed similar derangements in the follicular structure of the bowels to those previously discovered in several other diseases, and especially in that form of typhus fever now receiving the title of *dothinenteritis*. But there are cases, where, with some slight and weak phlogosis, the heaviest congestions are detected in nearly all the organs of the great cavities, and diverse effusions and extravasations are common events. These differences of appearance are referrible to the opposite conditions, which the disease presents in its most fatal forms.

Extensive sphacelation, in the anginose variety, is found in the fauces; and instances are mentioned in which it partially existed throughout the alimentary canal. Lately, some controversy has arisen as to the precise state of the throat, whether the appearances hitherto supposed to be sloughs from gangrenous inflammation, be really so, or are exudations of coagulate lymph, from the inflamed surface, imitative of sloughs. Either may undoubtedly exist. Deep ulcerations I have witnessed, and also, the coating of lymph, counterfeiting sloughs so accurately, as very readily to lead to the deception. There is here sometimes a perfect membrane, which, dipping into the larynx, has travelled down through the trachea into its final ramifications. This adventitious production, however, to such an extent, must be a rare occurrence, it being, at least, as I have seen it, uniformly restricted to the larynx, and much oftener in pieces, than as a whole lining of that structure. It varies from a mere pellicle, to a coating of considerable thickness and softness, even sometimes of a pultaceous consistence and aspect. These are the principal lesions hitherto noticed, in which, little peculiar or distinctive is to be remarked. Dissection, indeed, has as yet shed no light on the nature of scarlatina.

On this account, as well as from its analogy to those of the exanthemata of which I have treated, I mean to say not much concerning the pathology of this disease. Like its congenera, the primary impression, probably, is mainly on the primæ viæ, and the subsequent implication of other organs must be referred to a sympathetic or actual extension. The argument by which this view has already been sustained, in relation to the other cutaneous affections, might be applied with equal force to it. Many of the peculiarities, as well as the grades of violence of the disease, are owing to the difference of intensity in which its specific contagion operates. It is a poison, from the effects of which the system endeavours to extricate itself, and according to its resources, is the result. They being energetically applied, we have an open, inflammatory form of the disease; and conversely, a low congestive state, should they be feebly or incompetently exercised. Death, which is thought by many to be occasioned, in the anginose variety of it, by the state of the throat, does not seem to me to be justly assigned. This topical affection can rarely be productive of such an event, except when it is extended to the larynx. The disease is singularly pervading, characterized by great sensorial and nervous disturbance, and by an almost unexampled derangement, from inflammation or congestion, or both, of nearly all the important organs. It is by such lesions, that the constitutional powers become impaired, and ultimately extinguished. The throat suffers in common, runs into gangrene, from the loss of vitality, and perishes with the rest of the body.

In the cure of mild scarlatina, little more is required than the ordinary antiphlogistic regimen, watching at the same time, its tendency to those unfavourable changes formerly pointed out. None die of it, says Sydenham, except from too great officiousness in the practitioner. This may be too strongly affirmed, though it is still true, that the "nimia medici diligentia" here, as well as in the exanthematous fevers generally, is mischievous. Nature has established in all these cases a certain mode of relief, consisting in the exoneration in part, or wholly, of the internal tissues, by translating the irritation to the skin, and where she appears to be adequately effecting this end, it were better not to interfere with her endeavours. It is on such occasions, that the old maxim applies: "Nulla medicina, aliquando optima medicina." Certainly I have witnessed again and again, from the harrassing effects of active measures, the very worst consequences, converting what was originally mild, into a fearful degree of exasperation.

In the several presentations of the disease, however, either of a decidedly inflammatory or congestive character, the practice must be correspondently energetic, and in order to succeed, should be accommodated to these respective conditions. Let us first consider the course adapted to its phlogistic form.

The initiatory step depends on the stage of the case. Consulted early, or while there is rather irritation than positive inflammation, it will be well to commence with an emetic. It is highly commended by Tissot, Stoll, and the generality of writers; and I have reason to believe, proves exceedingly influensive in mitigating the future career of the case.

We are indeed told by Dr. Richard Harrison, an eminent practitioner of London, that the effect was so strikingly manifested in the disease during an epidemic prevalence of it in that city some years ago, that by many, the practice was reluctantly pursued, since the eradication of an attack was usually followed by another in a few days, and hence it was deemed better to postpone the emetic till the disease had taken such hold of the system as to do away any susceptibility to a future renewal of it.

My own observations, though decidedly in favour of this practice, do not corroborate this statement to the extent here made. Withering, however, who is one of the best writers on scarlatina, goes almost as far. "In the very first attack," says he, "a vomit seldom fails to remove the disease at once: if the poison has begun to exert its effects upon the nervous system, emetics stop its further progress, and the patients quickly recover. If it has proceeded still further, and occasioned that amazing action in the capillaries, which exists when the scarlet colour of the skin takes place, vomiting never fails to procure a respite to the anxiety, the faintness and delirium." But whatever difference of opinion may be entertained as to the general use of emetics, I think there can scarcely be any concerning their applicability to the anginose form of the disease, and especially when complicated with the affections of the windpipe or lungs, the most ample experience having demonstrated their singular adaptation to such cases.

Next in importance, are evacuations of the bowels by mild laxatives. Febrile excitement, however, being developed, and particularly when accompanied by any prominent topical affection, the loss of blood by venesection can no longer be postponed, and must be proportioned to the degree of the emergency.

Nevertheless, great difference of opinion prevails among writers as to the propriety of venesection. Most of those of the continent of Europe are in favour, while the preponderance of English authorities is opposed to it, as reducing strength without affording any relief. This is the language of Sims, Withering, Clark, and Willan, the latter of whom declares that he never saw a case of scarlatina in which blood-letting seemed to be indicated. Exceptions, however, exist among the English, to this condemnation of the practice. Moreton pursued it, Cullen seems not indisposed to it, and Armstrong enjoins it, where there is visceral inflammation.

Not a little of this contrariety of sentiment, in relation to the remedy, I presume, must be referred to its having been applied under opposite circumstances of the disease, and, of course, attended by very different results. Directed with discrimination, it cannot fail according to my experience, to be beneficial, and often even indispensable. Convinced of this, my own practice, indeed, is to bleed in nearly every case of undue excitement. Yet it is true, that the loss of blood has no direct curative tendency in the disease, it only abating action, without changing or subverting it, and as usually not as well borne to any great extent, it is not safe to detract it with the same freedom as in the more purely phlegmasial affections, or perhaps to the amount, that the existing indications in the case itself would seem to demand. Collapse, frightful, and sometimes even fatal, I have repeatedly seen to result from an abuse of the practice, and it is always hazardous in an advanced stage of the disease. Local affections merely, may be removed by leeches or cups.

More than in any other disease, is this state of scarlatina characterized by heat of surface, and here cold applications are obviously called for, and prove immensely serviceable. Either ablutions or sponging I have preferred. Not content, however, with these modes, some of the European practitioners contend for the superior efficacy of aspersions, or even immersions, in the coldest water. But these have always appeared to me as very rash expedients, and I have heard of the latter proving even fatal. By a gentleman* who graduated at Edinburgh some years ago, I was told that one of the Professors of that school, to show his entire confidence in the practice, tried it on one of his own children, who died while in the bath, by a sudden recession of the eruption—and a similar result, in another instance, is mentioned by Armstrong, from affusion only. Yet Currie informs us, that he was in the habit of stripping his patients, and dashing buckets of cold water on them, from which such benefit accrued, that the disease was frequently cut short in its progress.

Bateman commends this mode, as more efficacious in the very beginning, though subsequently in the disease, he thinks, every advantage may be attained by simply washing the surface.

Caution should certainly be practised in this and every other eruptive fever in the use of the remedy, from the danger of repercussion, and never ought it to be resorted to, without the strictest observance of those precepts formerly indicated for its regulation. Might not cold enemata, under such circumstances, and with similar restrictions, prove useful? They are so in other ardent fevers, and here, from the extraordinary warmth, both of the internal and external surfaces, would seem *a fortiori* to be exacted. But having no experience, I throw out the suggestion only as a plausible conjecture. From the great value I attach to cold applications, I cannot forbear further to urge their use, and more emphatically than I have done. Carefully accommodated to the case, they will, from what I have observed, invariably prove eminently serviceable, and very often indispensable to the cure. Many, truly, are the instances which I have witnessed, and some of them of considerable severity, where little else was required.

Not much is to be anticipated from the diaphoretic febrifuges at this period, which, owing to the state of the skin, rarely or never promote perspiration, or in any other mode reduce febrile excitement. Failing in the former respect, they are apt to be positively detrimental, by harrassing the internal surfaces, followed by general exasperation of condition; and such an effect being apparent, they

* Dr. Roper, of South Carolina.

should at once be discontinued. Yet it is of the highest importance, at this conjuncture, to restore the cutaneous functions: for, while these are suspended, it is impossible to overcome the disease, or to produce any sensible amendment. Not relieved, the skin, indeed, may have its vitality so far impaired, as never to be recovered, and in this mode, I believe, has often proved the cause of death, exactly as happens in small-pox, and other similar affections.

The depleting and evacuant remedies enumerated, as well as the cold applications, are calculated to effect this great end. But they are sometimes incompetent, and having been fairly tried without avail, other and opposite methods must be adopted, the best of which is tepid sponging, or immersion in moderately warm water, or the vapour bath, repeated from time to time, till coolness, relaxation, and softness of the integument, are induced. Even these, too, do not uniformly succeed, especially when too long postponed, or the skin is deeply affected; and in such an emergency, advantage might possibly be derived from some of those emollient applications employed in erysipelas, scalds, &c.

In the congestive variety of scarlatina, which is now to claim attention, the attack as formerly mentioned, is sometimes introduced by a very protracted collapse. The leading object here, is to arouse the recuperative powers, and to bring on due reaction, the means of effecting which having been detailed on several preceding occasions of essentially similar character, they need not again be recited with any particularity. It may be sufficient to remind you, that they consist of the warm bath, succeeded by frictions or sinapisms, or blisters, to the extremities, and above all, over the epigastrium, with warm beverages, or possibly some of the more active diaphoretics. Having attained the object in view, then an emetic, and afterwards purging with calomel, constitute the approved practice. These measures are held to be, by the highest authorities, as even more appropriate to this, than the other form of the disease. They are calculated to cleanse the primæ viæ, to prevent or remove congestions, and to excite the cutaneous surface.

To a greater extent, however, than any one else, was Hamilton addicted to the purgative practice. "Many years ago," says he, "when the prejudices against purgatives were more decided and prevalent than they are at this time, I continued to prescribe them. My doing so was, indeed, the necessary consequence of the advantage I had experienced from the same remedies in typhus. I had learnt, that the symptoms of debility which take place in this species of fever, so far from increasing, were obviously relieved by the evacuation of the bowels. I was, therefore, under little apprehension from them in scarlatina. I have never witnessed sinking or fainting, as mentioned by some writers, and so much dreaded by them: neither have I observed a revulsion from the surface of the body, and consequent premature fading, or in common language, the striking in of the efflorescence, from the exhibition of purgatives."

Considering how exceedingly vitiated are the secretions usually in this form of the disease, and the irritation which they must create when collected in the alimentary canal, the propriety of removing them seems scarcely disputable. Nevertheless, I suspect, the practice has been abused, as well by a too indiscriminate recurrence to it, as by urging it to excess. Let it be restricted to its legitimate purpose, and, I think, there can be no doubt of its safety and usefulness.

It is far less easy to decide on the expediency of venesection. The abstraction of blood appears to be required, by the loaded state of the organs, and contra-indicated by the depression of the vital energies. My own conviction is, that it should not be hazarded, unless reaction is pretty firmly established, the circulation in some force, and the skin warm—and, even here, is to be resorted to with circumspection. Much safer is topical bleeding by leeches or cups, and under equivocal circumstances, should be invariably preferred.

The case, however, becoming more decidedly typhoid, a resort must be had to a combination of calomel, opium, and ipecacuanha, repeated every hour or two. The mercurial practice of late so strenuously commended by some, originated in this country. It is upwards of a century since it was employed by Dr. Douglass, of Boston, in an epidemic scarlatina of that city, who extols the superior efficacy of it in a publication on the subject. Commencing with calomel as a purge, he next urged it to a salivation. The latter, however, in contradiction to his reports, I should think hazardous. It is better to aim only at its alterative effects; and such seems to be the end for which it is now, by common consent, directed. The difficulty, indeed, of salivating in this disease is very great, and there are some writers who deny that it ever takes place. No doubt it is among the rarest of occurrences, and I am inclined to suspect, that Douglass mistook the salivation, which is a common incident of the disease itself, for the mercurial effect.

But while thus conducting the general treatment, the affection of the throat, should it exist, must not be neglected. The best remedies for it in the beginning, are topical bleeding, blistering, and warm poultices.

Whatever may have been the precise character of the disease in the early stages, when exhaustion supervenes, there is considerable uniformity as to the plan to be pursued. An appeal is to be made to those means by which the resources of life are revived or strengthened; and of which carbonate of ammonia, camphor, wine whey, or wine itself, or even diluted ardent spirits, and opiates, if not especially contraindicated, are usually confessed to be the most efficacious. But the muriatic acid was at one time exceedingly confided in, having been introduced by Sir William Fordyce, who gave the strongest assurance of its efficacy, directing it with some bitter infusion, as that of the bark especially. By some of the West India practitioners, the compound infusion of capsicum, to be noticed presently, as a gargle, is considered also as among the very best of internal remedies, in the dose of a tablespoonful occasionally repeated. Nothing, however, have I found at this conjuncture, comparable to the sulphate of quinine, alone, or with laudanum, and the dulcified spirits of nitre, as may be indicated, aided by some cordial drink. Little is here gained by blisters or sinapisms. They rarely excite the skin; and when they do, the inflammation is very apt to degenerate into gangrene. But stimulating frictions are serviceable.

We must again revert to the anginose affection, which, changing with the progress of the disease, requires a modification of treatment. On the occurrence of the apthous appearance, mild detergent gargles are to be resorted to; and when sloughing commences, those of more activity, such as barley water with the sulphuric or muriatic acid, or a decoction of Peruvian bark with these acids, or the tincture of myrrh diluted, or an infusion of capsicum prepared in the following mode:

℞. Caps. coch. mag. ij.
Sodæ muriat. coch. min. iss.
Aq. bull. lb. ss.
Acid. acid. ib.
M. Infus. et collat.

It is probable that gargles of the chlorate of soda, or lime, might be beneficially used. But the best application I have tried is that of burnt alum, in a mode presently to be mentioned. The sloughs being detached, leaving an unhealthy surface, the black mercurial wash, or a solution of the sulphate of copper, or of the nitrate of silver, or preferably here, too, the burnt alum, may be applied by a small brush or hair pencil. But if, instead of ulceration, the fauces are covered by a membranous exudation, it is proposed for its removal, to apply to it in the same way the lunar caustic, or muriatic acid mixed with honey. Emetics are recommended in each case with a similar intention, or to cleanse the throat, and to institute a more healthy action. The sulphate of mercury has been praised particularly for this purpose, though I suspect it has no just claims to a preference. Nor are they less deserving of regard as emulgents of the bronchial structure when heavily oppressed by accumulations of viscid secretions, or from other matters.

This concludes the medical treatment of scarlatina in its several modifications.

Of regimen I have to observe, that it should harmonize with the remedies. The diet, in the decidedly inflammatory states, must consist of cold mucilaginous beverages exclusively, and if other drinks be urgently desired, which happens in very heated conditions, water may be allowed, of icy coldness, in small quantities. Even ice itself is admissible. Let, also, the freest ventilation be practised in hot weather, and the patient very slightly covered. But under other circumstances of the disease, the course is reversed. Either the eruption refusing to come out, or showing a disposition to recede, or the skin is cool, the pulse feeble, or any other evidence existing of typhoid prostration, a higher degree of temperature must be observed, the diet more nutritious, and the drinks warm, and, perhaps, cordial and exciting.

After recovery from the disease itself, there may be certain sequelæ or effects, to which our attention is often called. Cases are to be met with of enlargement of the parotids, or testicles, suppuration of the ears, various pectoral, or gastric, or enteric affections, hydropic effusion, or great derangement of the nervous system, expressed by palsy, chorea, hysteria, epilepsy, or neuralgia, as well as chronic cutaneous eruptions, mostly herpetic, and the developement of scrofula, &c. The most common, however, of these consequential lesions, is a hot, dry, harsh, unperspirable state of the skin, and especially when desquamation has not taken place. The functions of the surface, so essential to the order of health, are hence not performed, fever is kept up or revived, with a most harrassing degree of itching, preventive of all quietude or sleep, and chiefly occasioning, as I believe, most of those very affections I have enumerated, and particularly hydropic effusion. As the cause of so much mischief, this condition of the tegumentary tissue should always be removed without delay; and the most effectual means of doing it is tepid bathing, either by the sponge or immersion in a bath, taking special care to avoid any exposure to cold. Yet it may be necessary, where excitement is high, to bleed and purge moderately, and next resort to the mild diaphoretics. But from bad management or otherwise, dropsy is so very frequent an occurrence, and as it of the several affections alone exacts any peculiarity of treatment, it presents here an exclusive claim to consideration.

Generally it appears as edema of the lower extremities, or of the face and neck, though there are numerous instances of effusion, also, into the cavities. Bateman truly remarks, that when the anasarca becomes general, a sudden effusion occasionally takes place into the cavity of the chest, or into the ventricles of the brain, causing death in a few hours. Cases of this kind I have frequently seen.

Great contrariety of opinion prevails as to the management of this secondary dropsy. The fact is, that it is connected with such opposite states of the system, that no one plan is suited to all the cases. Existing, which usually happens, with an accelerated, hard, and febrile pulse, unrelenting skin, deficient urinary secretion, the little that passes away depositing a lateritious or pink-coloured sediment, there ought to be no hesitation as to the propriety of venesection, the saline purgatives, the mild diuretics, the nitrate of potash, or the cream of tartar especially, with a very low diet.

Treated differently, it invariably proves obstinate or fatal, of which Bursenius gives a very striking illustration. Early in the last century, an epidemic scarlatina prevailed at Florence, followed in nearly every case by more or less edema, pulmonary oppression, fever, and diminished urination. Tonics were for a time tried, under which the dropsy was greatly aggravated, and the mortality considerable. Dissection, however, disclosing inflammation of the lungs, alimentary canal, and kidneys, venesection with its auxiliaries was adopted, and the result came to be uniformly favourable. Yet it is no less true, that effusions may take place under circumstances directly the reverse of the preceding, or in a weak and highly cachectic condition. Moderate purging, and the more stimulating diuretics are here the remedies in which confidence is usually placed. But I have found an infusion of digitalis singularly efficacious, and in many instances so much so, as to supersede all other measures.

To restore strength and soundness of constitution, the martial preparations, exercise, and a nutritious diet, sometimes become necessary, and particularly in exsanguinous, or leucophlegmatic states. But where the treatment of the disease has been careful and judicious, such sequelæ are of rare occurrence, and may, perhaps, be entirely obviated.

As a preventive of scarlatina, the use of belladonna has, of late, attracted some attention. The facts, indeed, bearing on this point, are exceedingly curious, and coming from most respectable sources, ought not to be contemptuously or heedlessly passed over, or rejected. Berendt, a physician of Vienna, declares, that by the use of the article, only fourteen out of one hundred and ninety-five children exposed to the contagion took the disease, and these had it very mildly. We are further told by Professor Herholdt, of Copenhagen, that he found it to preserve several hundred children, during the prevalence of the disease as an epidemic in that city, and on a subsequent occasion, when it appeared even more violent, out of nearly a hundred families, all escaped except one, and of this, he is doubtful whether they took the medicine. The testimony of Dr. Korest, of Berlin, is scarcely less decisive; and a variety of attestations from other medical men of the continent of Europe might be advanced. Ten or fifteen drops, morning and night, of the watery solution of the extract of belladonna, in the proportion of two or three grains to the ounce, is the common mode of exhibition.

To what extent these statements are to be credited, I cannot say from any experience of my own. Distinct from the authority by which they are sustained, when we advert to the powerful impression of the belladonna, we may, on the principle of the incompatibility of two actions simultaneously existing, get an explanation of its modus operandi, and be not altogether incredulous. That pre-occupying the stomach by food, cordial drinks, and even by certain medicines, as opium, or bark particularly, has proved prophylactic as to intermittent and some other diseases, is abundantly demonstrated. Yet by the assurance of Hahnemann, the author of the homœopathic doctrines, which, founded in fraud and imposture, have alike deluded the credulous, and disgraced medicine, that he was equally successful in the infinitesimal dose of a few drops, daily, of a solution, each drop of which contained no more than the twenty millionth part of the grain of the extract, distrust, and even ridicule, are cast over the whole affair, and our faith must be withdrawn or suspended, till fresh and better evidence is afforded.

With this I conclude the account of those eruptive fevers, which, however they may differ in some particulars, are so nearly allied, by having one common source in a specific contagion, and running a definite career, reproductive of exactly the same sort of contagion, that, on the ordinary principles of classification, they seem to require to be separated from the other cutaneous affections, and to be arranged together as in one family.

Next I am to bring forward another set of these febrile diseases, which, though in their external physiognomy, they bear a close analogy to some of the former, are dependent on different causes, and in their nature are so dissimilar, that they must be placed under a different head.

